# Attention Deficit Hyperactivity Disorder Symptoms, Comorbidities, Substance Use, and Social Outcomes among Men and Women in a Canadian Sample

**DOI:** 10.1155/2015/982072

**Published:** 2015-05-03

**Authors:** Evelyn Vingilis, Patricia G. Erickson, Maggie E. Toplak, Nathan J. Kolla, Robert E. Mann, Jane Seeley, Mark vanderMaas, Deanne S. Daigle

**Affiliations:** ^1^Population and Community Health Unit, Department of Family Medicine, University of Western Ontario, 1151 Richmond Street, London, ON, Canada N6A 5C1; ^2^Department of Sociology, Centre for Criminology and Sociological Studies, University of Toronto, Toronto, ON, Canada M5S 2J4; ^3^Department of Psychology, York University, LaMarsh Centre for Child and Youth Research, Toronto, ON, Canada M3J 1P3; ^4^Complex Mental Illness, Forensic Service, Centre for Addiction and Mental Health, Department of Psychiatry, University of Toronto, Toronto, ON, Canada M5S 2S1; ^5^Centre for Addiction and Mental Health, Dalla Lana School of Public Health, University of Toronto, Toronto, ON, Canada M5S 2S1; ^6^Department of Sociology, Collaborative Program in Addiction Studies, University of Toronto, Toronto, ON, Canada M5S 2J4

## Abstract

*Background.* Attention deficit hyperactivity disorder (ADHD) is a neurodevelopmental disorder that can persist in adolescence and adulthood. *Aim.* To examine prevalence of ADHD symptoms and correlates in a representative sample of adults 18 years and older living in Ontario, Canada. *Method.* We used the Centre for Addiction and Mental Health Monitor, an ongoing cross-sectional telephone survey, to examine the relationships between ADHD positive symptoms and comorbidities, substance use, medication use, social outcomes, and sociodemographics. *Results.* Of 4014 residents sampled in 2011-2012, 3.30% (2.75%–3.85%) screened positively for ADHD symptoms (women = 3.6%; men = 3.0%). For men, distress, antisocial symptoms, cocaine use, antianxiety medication use, antidepressant medication use, and criminal offence arrest were associated with positive ADHD screen. For women, distress, cocaine use, antianxiety medication use, antidepressant medication use, pain medication use, and motor vehicle collision in the past year were associated with positive ADHD screen. *Conclusions.* ADHD symptoms are associated with adverse medical and social outcomes that are in some cases gender specific.

## 1. Introduction 

Attention deficit hyperactivity disorder (ADHD) is a neurodevelopmental disorder with symptoms of inattention, hyperactivity, and impulsivity that present in multiple settings [1]. Initially ADHD was viewed as a disease of childhood that declined or disappeared in adulthood. Research over the past 30 years has found ADHD to persist in adolescence and adulthood for 50% to 60% of childhood ADHD cases [2]; though ranges as extreme as 4% to 80% have also been reported [1–6].

Although symptoms of ADHD have been extended developmentally upward to adults and the diagnostic criteria of ADHD have been revised in the DSM-5 to reflect more accurately the experience of affected adults, adult ADHD research is in an early stage [1, 7, 8]. Most ADHD research has focused on homogeneous samples of clinically referred, young Caucasian males [9]. These samples have the advantage of extensive assessment but lack representation from nonclinical groups exhibiting ADHD symptoms [10, 11]. Clinical samples are found to show more symptoms and impairment [11]. Moreover, many of these studies suffer from major methodological shortcomings, including small sample sizes, referral biases, high loss to follow-up, inadequate matching of groups, and lack of gender inclusion and analysis [9, 10, 12]. On the other hand, population-based, representative samples overcome many of these methodological weaknesses, while their findings allow inferences to be made to the general population [11, 13].

Data on prevalence of adult ADHD are limited, but estimates based on international studies using multistage household probability samples range from 1.2 to 7.3% with an average of 3.4% [14]. No population-based adult ADHD prevalence estimates are available for Canada.

Current research suggests that gender differences in the prevalence of adult ADHD may differ from prevalence patterns reported in children. Studies of ADHD in children find that boys are much more affected than girls, with clinically referred studies having gender differences closer to 9 : 1 and epidemiological studies closer to 3 : 1 [15]. Among adults, results are mixed. Kessler et al. [16] found that diagnosis of ADHD in their survey of 18–44-year olds was 5.4% for men and 3.2% for women, while Faraone and Biederman [6] found no differences (men = 3.0%; women = 2.8%). Information on ADHD and age is very limited, despite the historical controversy on whether ADHD stays the same, declines, changes, or disappears in adulthood. A recent meta-analysis on adult ADHD found that only two studies included participants over 60 years of age; the mean ages of most studies were upper teens to mid-30s [9]. The meta-analysis of these age-limited studies showed a gender by age interaction with symptoms declining as men but not women reached their 40s.

Clinical studies of adolescents and adults with ADHD have found higher rates of psychopathology, such as mood, anxiety, childhood disruptive, antisocial personality, and substance use disorders compared with control groups [4, 5, 15, 17]. A recent meta-analytic study examining the association of childhood ADHD and substance use and abuse/dependence found that children with ADHD were significantly more likely as young adults to have ever used nicotine, cannabis, and cocaine but not alcohol and were significantly more likely to develop substance use disorders than controls [18].

Those with ADHD are also less likely to enter college and to graduate and generally have 2 years less schooling [19]. They are less likely to be employed and have lower SES and income and higher crash and criminal offence rates, although in some studies direct relationships between ADHD and various delinquencies disappear when comorbid conditions are included [2, 5, 20]. However, Weiss and Hechtman [3] found in their 15-year follow-up that although 50–60% of young adults initially diagnosed with ADHD continue to exhibit symptoms, their adult ADHD patients had lower risk of antisocial or criminal behaviours, despite slightly elevated rates in adolescence. Thus, there is a need for sound epidemiological data to understand the manifestations of ADHD symptoms in adulthood by gender, psychiatric comorbidity, and social outcomes.

The purpose of this study was to examine prevalence of ADHD symptoms and their relationship with comorbidities and social outcomes and to explore differences by gender, in a large population-based survey of adults in Ontario, Canada.

## 2. Methods

### 2.1. Sample

The data are based on telephone interviews (landlines and cell phones) with 4,014 Ontario adults (ages 18 or older) over 24 months between January 2011 and December 2012. The data are from the Centre for Addiction and Mental Health (CAMH) Monitor, an ongoing cross-sectional, computer-assisted telephone survey administered by the Institute for Social Research at York University, Canada (see [21] for details). Each monthly cycle uses a two-stage probability sampling procedure. In the first stage, a random sample of telephone numbers was selected with equal probability from within each regional stratum. In the second stage, one respondent aged 18 or older who was able to complete the interview in English was then selected from within each household according to the most recent birthday of all household members. Response rates based on estimated eligible sample averaged 52.89%.

### 2.2. Measures

All scales were based on well-validated measures and demonstrated good internal consistency.

#### 2.2.1. ADHD Measures

(1) Adult ADHD Self-Report Scale-V1.1 (ASRS-V1.1) was developed by Kessler et al. [22] as part of the WHO Composite Diagnostic Interview. Psychometric validation against DSM-IV based psychiatric diagnoses by experienced clinicians demonstrated that the 6-item, 5-point Likert scale screener was superior to the 18-item version on specificity (99.5% versus 98.3%), sensitivity (68.7% versus 56.3%), total classification accuracy (97.9% versus 96.2%), and Cohen's kappa (0.76 versus 0.58) [23–25]. Positive ADHD symptoms screen is a total score greater than 13 [25]. (2) Previous ADHD diagnosis was assessed by the item “have you ever been diagnosed with Attention Deficit Disorder (ADD) or Attention Deficit Hyperactivity Disorder (ADHD) by a doctor or health care professional?” (3) ADHD medication use was assessed by items querying if, when and how long, they had been treated with medication for ADHD or ADD by a doctor or health care professional, and whether they are currently taking it (adapted from Ontario Student Drug Use and Health Survey) [26].

#### 2.2.2. Psychiatric Distress and Medication Use Measures

(1) General Health Questionnaire (GHQ12) is a 12-item, 4-point widely used screening instrument for current psychiatric distress that captures depression/anxiety and problems with social functioning [27–29] with a score of three and higher as a positive screen; (2) depression/anxiety/pain medication use: in the past 12 months have you taken any prescription medication: to reduce depression? to reduce anxiety or panic attacks? for pain?

#### 2.2.3. Antisocial Behaviour Measure

(1) Antisocial Personality Disorder Scale from the Mini-International Neuropsychiatric Interview (MINI-APD), a 12-item, dichotomous scale, was designed to provide a short clinical screening tool to assess delinquencies (truancy, cheating/lying/stealing, bullying, and hurting animals/people) before and after age 15. We excluded one item of the MINI-APD (forced someone to have sex before age 15), as required by the ethics review board. A score of three or more on the latter six MINI-APD questions indicated a positive APD screen [30].

#### 2.2.4. Substance Use and Abuse Measures

(1) Lifetime cannabis and cocaine use (never used = 0, ever used = 1); (2) Alcohol Use Disorders Identification Test (AUDIT) is a validated screening instrument developed by the WHO, to detect individuals at the less severe end of the spectrum of alcohol problems, with a score greater than seven indicating hazardous alcohol use [31–33]. The AUDIT has been extensively used in both national and Ontario surveys which demonstrate the validity of the instrument in Canadian populations and the utility of the 8+ cutoff [34–36]. (3) The cannabis subscale of the Alcohol, Smoking and Substance Involvement Screening Test (ASSIST) is a 6-item screening instrument to assess risk of experiencing health and other problems (social, financial, legal, and relationship) from their current pattern of cannabis use, with scores of four or more indicating moderate or high risk of problems [37].

#### 2.2.5. Social Problems

These were self-report items: (1) vehicle crash involvement in past year and (2) ever in lifetime arrested for a criminal offence.

#### 2.2.6. Sociodemographics

(1) Gender, (2) age, (3) marital status, (4) educational status, (5) employment status, and (6) income were included.

### 2.3. Statistical Analysis

All analyses used linearized methods based on sample design and weighted by probability of selection. The percentages reported are considered representative for the population surveyed. We used logistic regression analysis to estimate odds ratios for ADHD positive symptoms associated with sociodemographics, previous ADHD diagnosis, medication use, comorbidities, substance use and abuse, and social problems. We conducted separate regression analyses for men and women. We adjusted for age, marital status, education, and employment. Results are based on “valid” responses; responses such as “do not know” and refusals were considered missing data and excluded from analyses.

## 3. Results

Overall, 3.30% (C.I.2.75%, 3.85%) of the sample screened above the cutoff for positive ADHD symptoms.  Table 1 indicates significant differences between those who screened positively and negatively for ADHD symptoms. A greater percent of those who screened positively were younger, less likely to be married, had lower education, and were part-time employed or other, compared to those who screened negatively. A higher percentage of those who screened positively for ADHD symptoms reported previous ADHD diagnosis, psychiatric, substance use, and social problems compared to those who screened negatively.

Examination of sociodemographics by gender indicates (Table 2) that for men and women the odds of screening positively for ADHD symptoms were significantly lower for the 25–44-year-old age group compared to the 18–24-year-old group. However, women aged 45–64 had significantly lower rates than those aged 25–44, and those aged 65 and older showed lower rates than those aged 45–64. For marital status, no differences were found by gender.

Different patterns emerged between men and women on education and employment. Among men, those who reported not completing postsecondary education showed higher odds of screening positively for ADHD symptoms than those with only a high school education. For women, those who reported achieving a postsecondary diploma or degree showed significantly lower odds of screening positively for ADHD symptoms than those who did not complete a postsecondary degree. For employment no significant differences were observed among men between those who reported being unemployed and those who reported working either full or part time. Among women, however, those who reported being employed full time showed significantly lower odds of screening positively for ADHD symptoms.

The adjusted logistic regression (Table 3) shows variables with significant odds ratios. Those who reported previous diagnosis for ADHD and ADHD medication use showed significantly higher odds of screening positively for ADHD symptoms. Gender differences were also found: women showed much higher odds of screening positively for ADHD symptoms when they had been diagnosed (adjusted odds ratio [OR] 15.04, 95% confidence interval [CI] 6.40–35.25) or treated for ADHD (OR 19.25, CI 7.47–49.61) compared to men who reported being diagnosed (OR 6.14, CI 2.65–14.24) or treated (OR 9.00, CI 3.73–21.70). The results also indicated that psychiatric symptoms and medications were comorbid with ADHD positive symptoms. Those who screened positively on the GHQ for distress and reported antidepressant and antianxiety medication use had higher odds of screening for ADHD symptoms. Women also showed significantly higher odds of screening for ADHD symptoms when they reported using prescription pain killers in the last year, while men showed no significant relationship. Both men and women who reported past year cannabis use and cocaine use ever showed significantly higher odds of screening positively for ADHD symptoms.

Gender differences also emerged for social problems. While women who reported a collision in the last year showed significantly higher odds of screening positively for ADHD symptoms, no significant relationship was found between arrest for criminal offence and ADHD symptoms. However, the findings were reversed for men.

## 4. Discussion

This first population-based Canadian study of adult ADHD symptoms found prevalence of 3.3% screening positively for ADHD symptoms and no differences between men and women. This is congruent with M. D. Weiss and J. R. Weiss's [37] suggestion that the higher clinical prevalence of boys to girls could reflect differential referral patterns between children and adults. This finding is also consistent with the average prevalence of 3.4% found in a review of other countries using similar epidemiological sampling methodology [13]. Kessler et al. [25], using the same ADHD screening instrument, found 4.4% screened positively for ADHD, but their sample was limited to American adults 18–44 years of age. Our study is among the few that included elderly persons. We found reported ADHD symptoms highest among the 18–24-year olds, consistent with other studies [38], although for women the odds of ADHD positive symptoms decreased across all age groups whereas for men ADHD positive symptoms stabilized from young adulthood. Nonetheless, our results confirm that ADHD symptoms persist for some adults across the adult lifespan among both men and women and are associated with adverse psychiatric, substance use, and social outcomes that were in some cases gender specific. Accordingly, these results suggest the importance of screening for ADHD among adult patients presenting with internalizing or externalizing psychiatric symptoms and/or substance use.

Our study found few differences in the clinical phenotypes of men and women who screened positively for ADHD symptoms. Although diagnosis of ADHD among those who screened positively was low at 22.7%, women reporting ADHD diagnosis and medication treatment had more than two times higher adjusted odds of screening positively for ADHD symptoms compared to men. Given that girls with ADHD tend to be underidentified because of their less disruptive behaviours [37], it may be that those who have been diagnosed have more severe symptoms and hence are more likely to have been treated. Additionally, it may be that women are more willing to respond positively to screening items compared to men.

Psychiatric comorbidities are commonly found among adult ADHD patients, in particular, mood, anxiety, substance use, and antisocial personality disorders [5, 37]. The higher adjusted odds of positive ADHD symptoms for those who reported distress and antidepressant and antianxiety medication use could reflect the higher comorbidities found with ADHD. A portion could also be due to misdiagnosis of ADHD symptoms or possibly treatment with SSRIs for ADHD. Or ADHD symptoms can also be part of the symptom cluster for mood and anxiety disorders.

ADHD patients are also at significant risk for substance use, particularly for cannabis and cocaine, with 40% prevalence of lifetime diagnosis of substance use disorders [39]. A recent meta-analysis found that young adults diagnosed with ADHD as children were significantly more likely to use cannabis and cocaine but not alcohol [18]. Our study found that for those screening positively for ADHD symptoms, adjusted odds for cannabis use in past year and lifetime cocaine use were significantly higher, but not for the AUDIT or the ASSIST suggesting greater use but not necessarily abuse. The gender differences found for prescription pain medication, antisocial screen, and collisions in past year and lifetime arrest for criminal offence may reflect differences in externalizing and internalizing outcomes for men and women associated with ADHD symptoms.

The results of this study are subject to important limitations. These data are based on self-report screeners and do not reflect the breadth of information needed for clinical diagnoses. This is a key issue because the current study only reflects self-reported symptoms and does not examine impaired functioning and other issues related to specific diagnoses. Indeed, Gambino [40] cautions on the use of screening tests in prevalence estimation because screening tests tend to have relatively high false positive rates which result in overestimation of true prevalence in a population study. Thus, this population based sample may represent functioning persons with some ADHD and/or other comorbid symptoms but not actual diagnoses, although the screening tool (ASRS-V1.1) was validated against DSM-IV based psychiatric diagnoses by experienced clinicians and demonstrated good psychometrics. Additionally, although the response rate over 50% is very good for a telephone survey and data were weighted to reflect a representative sample of Ontario residents, the sample could potentially be biased. Moreover, although the total sample size is over 4000, cell sizes can be very small because psychiatric problems, such as ADHD, substance use, and collisions have low prevalence; small cell sizes and large CIs suggest a low level of precision, as indicated in some of the variables in Table 3. Nevertheless, these observations are of substantial interest in providing prevalence estimates by gender and by age, particularly for older age groups.

There are several clinical implications. Higher reports of ADHD symptoms are associated with more difficulties, further validating the ADHD self-report construct in a community sample. The relationships between the variables in this study also provide insights into better operationalizing self-reported adult ADHD symptoms. ADHD is typically regarded as an externalizing condition, but it has been acknowledged in the literature that internalizing symptoms are comorbid in adolescence and adulthood [6]. It is unclear why these internalizing symptoms emerge, whether they emerge biologically after adolescence or whether adults with ADHD have become depressed because of their chronic behavioural difficulties that may have persisted since childhood. The nature of the comorbidities is important to understand for treatment directions. A final notable finding is the proportion of symptoms reported in the 18–24-year olds that is higher than that in the older participants. This will be an important finding to follow to determine whether this is the result of more public awareness of ADHD, or whether this reflects some cohort effect in increased reported ADHD symptoms in our young people.

## Figures and Tables

**Table 1:** Characteristics of CAMH Monitor survey respondents who screened positively and negatively for symptoms on the Adult ADHD Self-Report Scale-V1.1 January 2011–December 2012.

	ADHD +		ADHD −		*P*
	Screen		Screen		
	*N*	%	*N*	%
Age					
18–24	26	5.4	458	94.6	<0.001
25–44	54	3.9	1346	96.1	
45–64	49	3.6	1326	96.4	
≥65	4	0.6	666	99.4	
Gender					
Female	76	3.6	2023	96.4	0.254
Male	57	3.0	1847	97.0	
Marital status					
Married/partner	70	2.6	2584	97.4	0.002
Widowed/sep./div.	18	4.1	421	95.9	
Never married	44	5.0	831	95.0	
Education					
High school	20	5.2	366	94.8	0.015
Completed HS	20	2.5	791	97.5	
Some after sec.	56	3.9	1377	96.1	
Univ. deg.	33	2.5	1304	97.5	
Employment status					
Full time	54	2.8	1903	97.2	0.018
Part time	22	5.5	380	94.5	
Other	56	3.4	1586	96.6	
Household income					
<20,000	11	8.3	121	91.7	<0.001
20–49,999	31	15.4	579	94.9	
50–100,000	39	19.0	1042	96.4	
>100,000	31	2.6	1152	97.4	
ADHD previously diagnosed					
Yes	22	22.7	75	77.3	<0.001
No	111	2.9	3783	97.1	
ASPD screen (≥4)					
ASPD	10	25.6	29	74.4	<0.001
Non-ASPD	121	3.1	3791	96.9	
Psychiatric distress (GHQ-12)					
Yes (+3)	67	11.8	502	88.2	<0.001
No (0–2)	66	1.9	3368	98.1	
Ever treated with ADHD meds					
Yes	6	15.0	34	85.0	<0.001
No	62	3.3	1802	96.7	
Antidepressant meds use					
Yes No	48 85	17.5 2.3	227 3633	82.5 97.7	<0.001
Anti-anxiety Meds Use					
Yes	27	19.6	111	80.4	<0.001
No	42	2.4	1725	97.6	
Pain Meds Use (with and without pres.)					
Yes	47	5.3	843	94.7	<0.001
No	85	2.8	2996	97.2	
AUDIT					
Yes (8+)	25	5.0	477	95.0	0.039
No (0–7)	106	3.1	3295	96.9	
ASSIST					
Mod/High (4+)	15	7.7	180	92.3	<0.001
Low (0–3)	116	3.1	3678	96.9	
Lifetime used cannabis					
Yes	94	5.8	1520	94.2	<0.001
No	38	1.6	2329	98.4	
Past 12 mths cannabis use					
Yes	38	7.3	486	92.9	0.008
No	94	2.7	3352	97.3	
Lifetime used cocaine					
Yes	32	11.3	251	88.7	<0.001
No	100	2.7	3606	97.3	
Ever arrested for a crime?					
Yes	23	7.5	284	92.5	<0.001
No	110	3.0	3573	97.0	
Crash in past year					
Yes	13	6.0	202	94.0	0.03
No	120	3.0	3868	97.0	

**Table 2:** Odds ratios (ORs) and confidence intervals (CIs) for positive ADHD symptoms by sociodemographic variables for men and women.

Variables	Men			Women		
	OR	95% CI	*P*	OR	95% CI	*P*
Age						
18–24 (ref.)						
25–44	0.18	0.06–0.53	^**^	0.13	0.02–0.64	^∗^
45–64	0.86	0.36–2.03	NS	0.12	0.03–0.40	^***^
≥65	0.83	0.47–1.47	NS	0.51	0.28–0.95	^∗^
Marital status						
Married (ref.)						
Wid./div./sep.	2.20	0.84–5.77	NS	1.91	0.96–3.79	NS
Never married	1.12	0.44–2.80	NS	1.41	0.73–2.74	NS
Education						
<high school (ref.)						
High school compl.	0.59	0.33–1.04	NS	0.55	0.28–1.06	NS
Some after sec.	2.1	1.15–3.65	^∗^	0.93	0.52–1.66	NS
University degree	0.88	0.48–1.59	NS	0.51	0.31–0.83	^**^
Employment						
Unemployed (ref.)						
Full time	0.82	0.41–1.67	NS	0.57	0.33–0.98	^∗^
Part time	2.17	0.94–4.99	NS	0.91	0.46–1.78	NS

NS: not significant, ^∗^ *P* < 0.05, ^∗∗^ *P* < 0.01, and ^∗∗∗^ *P* < 0.001.

**Table 3:** Adjusted odds ratios (ORs) and confidence intervals (CIs) associated with positive ADHD symptoms for men and women by ADHD diagnosis, medication use, comorbidity screeners, and social problems as relevant in overall regression analyses.

Variables	Men			Women		
	OR_adj_	95% CI	*P*	OR_adj_	95% CI	*P*
ADHD diagnosis	6.14	2.65–14.24	^***^	15.04	6.40–35.35	^***^
ADHD meds	9.00	3.73–21.70	^***^	19.25	7.47–49.61	^***^
Presc. pain meds	1.28	0.68–2.39	NS	2.02	1.24–3.28	^**^
Antianxiety meds	4.88	2.38–10.02	^***^	9.07	5.47–15.06	^***^
Antidepressant meds	6.59	3.13–13.89	^***^	8.75	5.17–14.79	^***^
Distress—GHQ	6.48	3.59–11.70	^***^	5.31	3.26–8.66	^***^
Antisocial screen (≥4 symptoms)	11.61	4.01–33.63	^***^	No cases		
Cannabis use Past yr	2.24	1.20–4.20	^∗^	2.20	1.18–4.08	^∗^
Cocaine use Lifetime	3.40	1.73–6.64	^***^	4.51	2.32–8.75	^***^
Collision Past yr	0.36	0.05–2.46	NS	2.97	1.43–6.19	^**^
Arrested criminal offence lifetime	2.62	1.33–5.16	^**^	1.25	0.42–3.67	NS

OR_adj_: odds ratio adjusted for age, marital status, education, and employment status, NS: not significant, ^∗^ *P* < 0.05, ^∗∗^ *P* < 0.01, and ^∗∗∗^ *P* < 0.001.
